# Evaluation of Biological Effective Dose in Gamma Knife Staged Stereotactic Radiosurgery for Large Brain Metastases

**DOI:** 10.3389/fonc.2022.892139

**Published:** 2022-06-30

**Authors:** Taoran Cui, Joseph Weiner, Shabbar Danish, Anupama Chundury, Nisha Ohri, Ning Yue, Xiao Wang, Ke Nie

**Affiliations:** ^1^ Rutgers Cancer Institute of New Jersey, Rutgers, The State University of New Jersey, New Brunswick, NJ, United States; ^2^ Jersey Shore University Medical Center (JSUMC), Neptune, NJ, United States

**Keywords:** gamma knife (GK), stereotactic radiosurgery (SRS), biological effective dose (BED), large brain metastases, staged radiosurgery

## Abstract

**Objective:**

Gamma knife (GK) staged stereotactic radiosurgery (Staged-SRS) has become an effective treatment option for large brain metastases (BMs); however, it has been challenging to evaluate the total dose because of tumor shrinkage between two staged sessions. This study aims to evaluate total biological effective dose (BED) in Staged-SRS, and to compare the BED with those in single-fraction SRS (SF-SRS) and hypo-fractionated SRS (HF-SRS).

**Methods:**

Patients treated with GK Staged-SRS at a single institution were retrospectively included. The dose delivered in two sessions of Staged-SRS was summed using the deformable image registration. Each patient was replanned for SF-SRS and HF-SRS. The total BEDs were computed using the linear-quadratic model. Tumor BED_98%_ and brain V_84Gy2_, equivalent to V_12Gy_ in SF-SRS, were compared between SF-SRS, HF-SRS, and Staged-SRS plans with the Wilcoxon test.

**Results:**

Twelve patients with 24 BMs treated with GK Staged-SRS were identified. We observed significant differences (*p* < 0.05) in tumor BED_98%_ but comparable brain V_84Gy2_ (*p* = 0.677) between the Staged-SRS and SF-SRS plans. No dosimetric advantages of Staged-SRS over HF-SRS were observed. Tumor BED_98%_ in the HF-SRS plans were significantly higher than those in the Staged-SRS plans (*p* < 0.05). Despite the larger PTVs, brain V_84Gy2_ in the HF-SRS plans remained lower (*p* < 0.05).

**Conclusion:**

We presented an approach to calculate the composite BEDs delivered to both tumor and normal brain tissue in Staged-SRS. Compared to SF-SRS, Staged-SRS delivers a higher dose to tumor but maintains a comparable dose to normal brain tissue. Our results did not show any dosimetric advantages of Staged-SRS over HF-SRS.

## Introduction

Brain metastases (BMs) have been reported in approximately 40% of patients diagnosed with cancer. Whereas whole brain radiotherapy (WBRT) has been traditionally offered for the treatment of BMs, stereotactic radiosurgery (SRS) has been shown as an alternative treatment modality with a similar local control rate and long-term survival but reduced neurocognitive toxicities compared to WBRT. Despite the excellent management of small BMs using single-fraction SRS (SF-SRS), the local control rates of BMs larger than 2 or 3 cm in diameter have been inferior (1, 2). As suggested in the RTOG 90-05 protocol (3), BMs measuring 2–3 cm or larger than 3 cm are treated to 18 Gy and 15 Gy, respectively, in order to reduce radiation dose spillage to the normal brain tissue and therefore minimize the risk of neurological toxicity, comparing to 20–24 Gy prescribed to smaller BMs measuring less than 2 cm. However, the administration of these lower prescription doses delivers lower biological effective dose (BED) and inevitably resulted in the suboptimal local control rate of large BMs.

To address the modest local control rate of large BMs, multiple strategies have been developed using hypo-fractionated SRS (HF-SRS) (4–7) and staged SRS (Staged-SRS) (8–14) to escalate prescription dose with promising results. Both hypo-fractionated and staged approaches take the advantage of the different radiobiological responses to radiation between tumor and normal tissues by distributing the radiation dose over a period, so that the repair of sublethal damage in normal tissue is allowed to reduce radiation-induced toxicities, while the same BED used in SF-SRS can be delivered to BMs to achieve optimal local control rate. The treatment schedules, however, differ between the two approaches. HF-SRS is usually delivered in 3–6 fractions over less than 2 weeks using a single treatment plan. Staged-SRS usually consists of 2–3 sessions separated by 2- to 4-week intervals where an individual plan is generated for each session due to the possible anatomy change during the long interval between the sessions. The linear-quadratic (LQ) model widely utilized in conventional external beam radiotherapy is readily applicable for HF-SRS since the treatment schedules of HF-SRS are similar to those used in external beam radiotherapy. However, due to the anatomical variation between Staged-SRS sessions and the tumor cell repopulation process during the interval, the simple summation of the BED delivered in both sessions is no longer valid to evaluate the radiobiological responses of tumor and normal tissue. Therefore, the purpose of this study was to generate a dedicated workflow to evaluate the BEDs to tumor and normal tissue in Staged-SRS accounting for possible tumor shrinkage and repopulation between the sessions, and to compare the radiobiological response of tumor and normal tissues between Staged-SRS, HF-SRS, and SF-SRS.

## Methods

### Clinical Workflow

Patients treated for large BMs using 2-staged gamma knife (GK) SRS at our institution were retrospectively identified for this IRB-approved study [this IRB approved study (Pro2018000227)]. The treatment protocol of Staged-SRS at our institution is similar to those that were previously reported (10, 13, 14). Briefly, two consecutive Staged-SRS treatment sessions with a 4-week interval were administrated to any targets of maximum diameter larger than 2 cm or total volume larger than 4 cc. If any other smaller lesions were also identified during the imaging study of each Staged-SRS session, they were treated concurrently with the SF-SRS technique following the RTOG 9508 protocol (15).

Per our institutional protocol, a series of T1- and T2-weighted MRI were acquired for each Staged-SRS session. Gross tumor volumes (GTVs) of BMs were contoured as radiographical enhancements on contrast-enhanced T1-weighted FSPGR MRI in 1.5-mm axial cuts and verified on an independent series of contrast-enhanced T1-weighted MRI coronal cuts. Patients were immobilized with frame fixation on the treatment day. Neither clinical nor setup margin was used; therefore, planning target volumes (PTVs) were identical to GTVs.

For each Staged-SRS session, an independent treatment plan was generated using the Elekta GammaPlan treatment planning system with a prescription dose of 12–15 Gy delivered to the 40%–60% isodose line. Shot positions and weights in the treatment plans were manually adjusted to achieve 100% target coverage and >0.6 target selectivity. The treatment plans were reviewed and approved by both radiation oncologists and neurosurgeons before the delivery.

### Dose Summation

**Figure 1:**
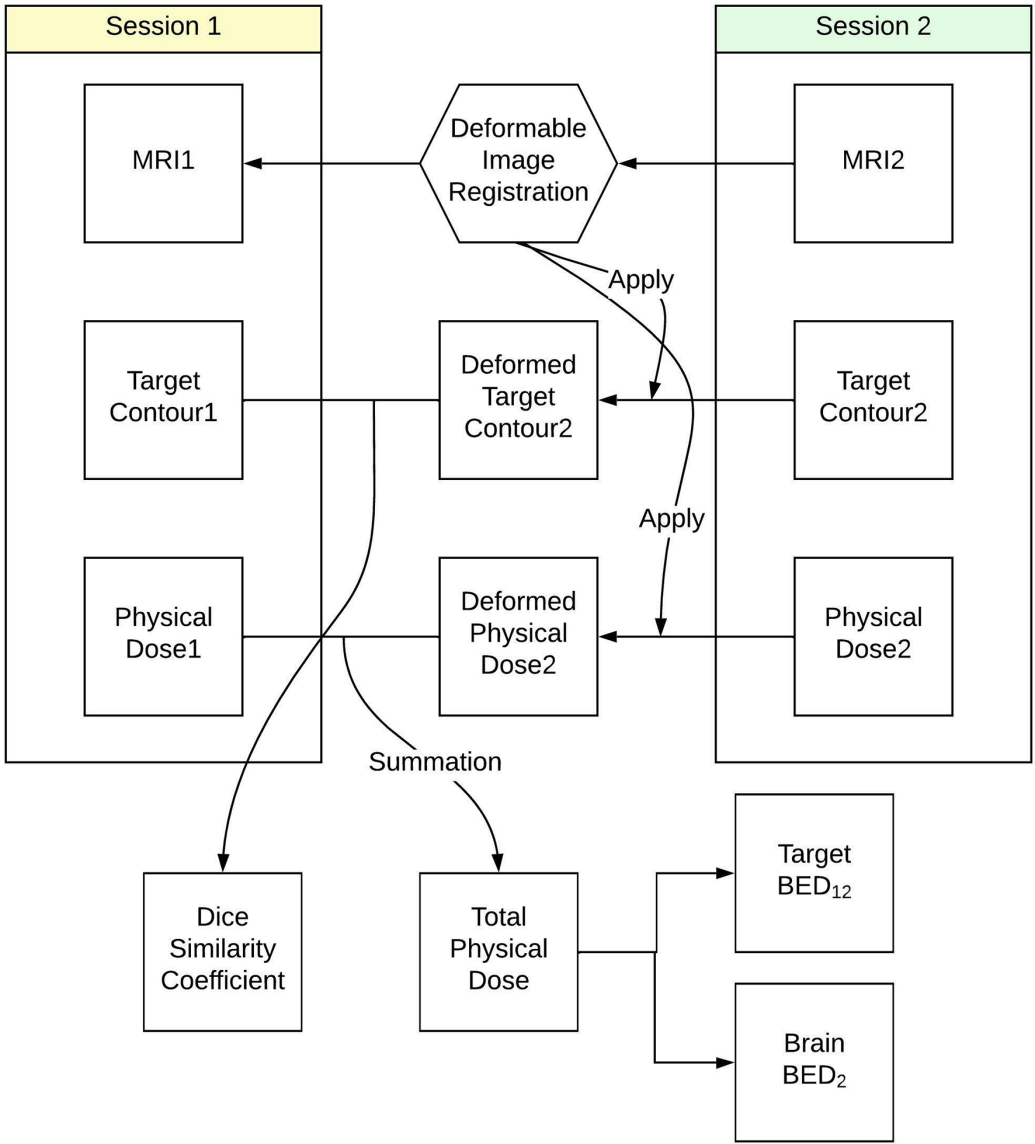
Workflow to calculate total biological effective dose delivered to tumor and normal brain tissue in Staged-SRS.

The workflow used to calculate the total BED delivered to tumor and normal brain tissue is summarized in **Figure 1**. To evaluate the accumulated dose delivered to tumor and normal brain tissues in both Staged-SRS sessions, we first performed a deformable image registration (DIR) between the T1-weighted MRIs acquired in the first and second sessions using a commercial software Velocity AI (Varian, Palo Alto, CA). Due to the possible variations in tumor volumes and patient setups between the sessions, the traditionally used rigid image registration was not applicable since the assumption of invariant patient anatomy was no longer held. The workflow of deformably registering the second MRI to the first MRI is detailed as follows: A cubic volume of interest (VOI) was first defined to encompass radiographically enhanced tumors and surrounding normal brain tissues. The voxels inside the VOI in the second MRI were deformably registered to those in the first MRI based on their intensity values; therefore, the original tumor in the first MRI matched with the shrunk one in the second MRI, as well as the sulci and gyri in the vicinity of the tumor. A deformation vector field, which correlates the corresponding voxels between the MRIs, was then applied to deform the GTV contour defined on the second MRI. The deformed GTV contour on the second MRI was then compared with the original GTV contour defined on the first MRI. The results of DIR were visually examined by a medical physicist for registration accuracy. The DIR was repeated if any large discrepancy was observed between the deformed and original contours, or any anatomically unsound deformations were found. Once the accuracy of DIR was satisfactory, the Dice similarity coefficient was calculated between the deformed and original contours to quantitively evaluate the performance of DIR. For a perfect DIR, the deformed and original GTV contours should be identical, and the Dice similarity coefficient is unity. The same deformation vector field was applied to deform the dose distribution in the second session, which could be combined with the dose distribution of the first session for dose summation.

The deformed dose distribution of the second session and the original dose distribution of the first session were exported into Matlab (MathWorks) as three-dimensional matrices, where the value of each voxel represented the physical dose delivered to that voxel. The original GTV contour in the first session was superimposed to both dose distributions to differentiate the tumor from the normal brain tissues. BED delivered to each voxel was calculated using Eq. 1 following the LQ model to describe cell survival during radiotherapy (16–18).


(1) $${\text{BED}}^{\text{i}}=D^{i}\left(1+\frac{D^{i}}{\alpha /\beta \;}\right)$$

Where *D^i^* is the physical dose delivered to either tumor or normal brain tissues in the *i*th Staged-SRS session. The *α*/*β* ratio in the equation is used to model the repair capability of a certain type of cell. An *α*/*β* ratio of 12 Gy was used for BMs based on a previous study on the treatment of large BMs using SRS (19), and a ratio of 2 Gy was used for normal brain tissue (20).

Whereas cell repopulation is usually ignored in the LQ model for conventional external beam radiotherapy or HF-SRS due to the relatively shorter radiation delivery time and a shorter interval between two consecutive deliveries compared to cell repair time, because of the considerably longer treatment interval between two Staged-SRS sessions, tumor cell survival would be undesirably increased due to cell repopulation. In order to reflect the potential decrease in tumor local control, a subtractive repopulation factor (17, 21) was included in the calculation of BED to account for potential tumor proliferation during the intentional interval. Therefore, the total BED delivered to a tumor voxel of a complete Staged-SRS treatment course could be calculated as


(2) $${\text{BED}}_{12}=D^{1}\left[1+\frac{D^{1}}{{\left(\alpha /\beta \right)}_{12}\;}\right]+\;D^{2}\left[1+\frac{D^{2}}{{\left(\alpha /\beta \right)}_{12}\;}\right]-\frac{\ln2}{\alpha T_{pot}}\text{max}\left(0,T-T_{k}\right)$$

Where *T_pot_* is the tumor potential doubling time, *T_k_* is the repopulation kick-off time, and *T* is the interval between Staged-SRS sessions. Given the nature of fast proliferating tumors, we assumed similar rapid repopulation rates for the cases included in the study and used the estimated values *a* = 0.3 Gy^-1^ (22), *T_pot_* = 3 days, and *T_k_* = 28 days (23, 24) in Eq. 2.

Since cell repopulation is negligible for late responding normal tissue, the total BED to a normal brain tissue voxel is simply the summation of the dose in both sessions and could be expressed as


(3) $${\text{BED}}_{2}=D^{1}\left[1+\frac{D^{1}}{{\left(\alpha /\beta \right)}_{2}\;}\right]+\;D^{2}\left[1+\frac{D^{2}}{{\left(\alpha /\beta \right)}_{2}\;}\right]$$

### Comparison Between Staged-SRS, SF-SRS, and HF-SRS

SF-SRS and HF-SRS plans were generated for each case to compare the BED delivered to both tumor and normal brain tissue. Assuming tumors were treated in a single fraction with frame fixation and the prescription dose following the RTOG 9508 protocol (15), SF-SRS plans were created by re-normalizing the prescription dose to tumors in the first session of the original Staged-SRS plans. In addition, given that HF-SRSs were usually delivered with frameless fixation, a 1-mm setup margin accounting for intrafraction motion was added to each original tumor to create a PTV. HF-SRS plans were manually created to prescribe 30 Gy in 5 fractions to each PTV. Both SF-SRS and HF-SRS plans met the institutional planning guidelines (target coverage > 100% and selectivity > 0.6) and were reviewed by radiation oncologists and neurosurgeons. Because no tumor volume change or tumor repopulation was considered, the BEDs to tumor and normal brain tissue were calculated using Eq. 1 for both SF-SRS and HF-SRS plans.

The minimum BED delivered to at least 98% (BED_98%_) of each tumor and the volume that received at least 84 Gy_2_ (V_84Gy2_) of normal brain tissue were compared between three plans. The BED_98%_ of GTV has been shown as a predictor of tumor local control rate for SRS (25), and the V_84Gy2_ of the normal brain tissue, which is biologically equivalent to V_12Gy_ assuming an *α*/*β* of 2 Gy, has been used to evaluate the risk of radiation-induced toxicity for SRS (26, 27). To quantitively compare the plan qualities between SF-SRS, HF-SRS, and Staged-SRS plans, the paired Wilcoxon rank sum tests were performed for statistical analysis with a significance level of *p* < 0.05.

## Results

Twelve patients treated with GK Staged-SRS for a total of 24 BMs were retrospectively identified for the study. The primary diagnoses were breast cancer (*n* = 6), non-small cell lung cancer (NSCLC) (*n* = 4), and melanoma (*n* = 2). The median age of patients at the time of first session was 59.5 years (range: 29–80 years). The majority of patients were female (*n* = 10). Of a total of 12 patients, only one had received WBRT prior to the Staged-SRS. The mean ECOG score before the treatment was 1 (range: 0–3).

Of the 12 patients, 3 (25.0%) had 4 lesions that received Staged-SRS, 1 (8.3%) had 3 treated, and 8 (66.7%) had 1 treated. The median interval between the two sessions was 30.5 days (range: 21–51 days). The median prescription dose for the first and second sessions was 13 Gy (range: 13–15 Gy) and 13 Gy (range: 12–13 Gy), respectively. Of the 24 lesions, 23 had a tumor volume reduction prior to the second session. The median tumor volume before the first and second sessions was 9.58 cc (range: 4.26–20.92 cc) and 4.30 cc (range: 0.78–17.80), respectively. The median relative tumor volume reduction was −52.2% (range: −81.8% to 4.3%), and this reduction was statistically significant (paired Wilcoxon; *p* < 0.005). The characteristics of patients and BMs are summarized in **Table 1**.

**Table 1:** Characteristics of patients selected in the study.

Patient	Primary	Age	Gender	Prior WBRT?	ECOG	Interval (days)	First Session			Second Session		
							Rx Dose (Gy)	Tumor Volume (cc)	Diameter (cm)	Rx Dose (Gy)	Tumor Volume (cc)	Diameter (cm)
1	Melanoma	68	F	No	1	26	13	7.92	2.46	13	3.6	1.59
2	Breast	57	F	Yes	2	41	13	9.95	3.11	13	4.78	2.61
3	NSCLC	61	F	No	0	42	13	7.24	2.67	13	2.32	1.84
4	Breast	68	F	No	3	26	13	13.12	4.15	13	6.7	2.77
							13	9.46	3.26	13	4.83	2.16
							13	7.72	2.9	13	3.48	1.96
5	Melanoma	58	M	No	3	21	13	4.02	2.05	12	2.52	1.48
6	NSCLC	79	F	No	2	51	13	17.07	3.6	13	17.8	3.19
							13	12.72	3.52	13	2.83	2.18
							13	9.44	2.85	13	4.51	2.1
							13	17.36	3.47	13	10.92	2.8
7	Breast	44	F	No	0	21	13	16.18	4.31	13	7.59	3.45
							13	12.45	2.92	13	4.8	2
							13	10.37	3.16	13	5.7	2.64
							13	7.64	2.77	13	3.05	1.92
8	Breast	29	F	No	1	33	13	12.83	3.17	13	4.08	2.2
							13	8.43	2.4	13	4.3	1.99
							13	4.8	2.22	13	0.93	1.45
							13	4.26	2.76	13	0.78	1.16
9	NSCLC	83	F	No	2	46	13	16.55	3.18	13	12.44	2.74
10	Breast	54	F	No	1	28	15	6.9	2.78	13	3.64	2.05
11	NSCLC	80	M	No	1	28	13	20.92	4.17	13	13.2	3.05
12	Breast	48	F	No	1	42	13	9.58	2.72	13	4.23	2.02

**Figure 2:**
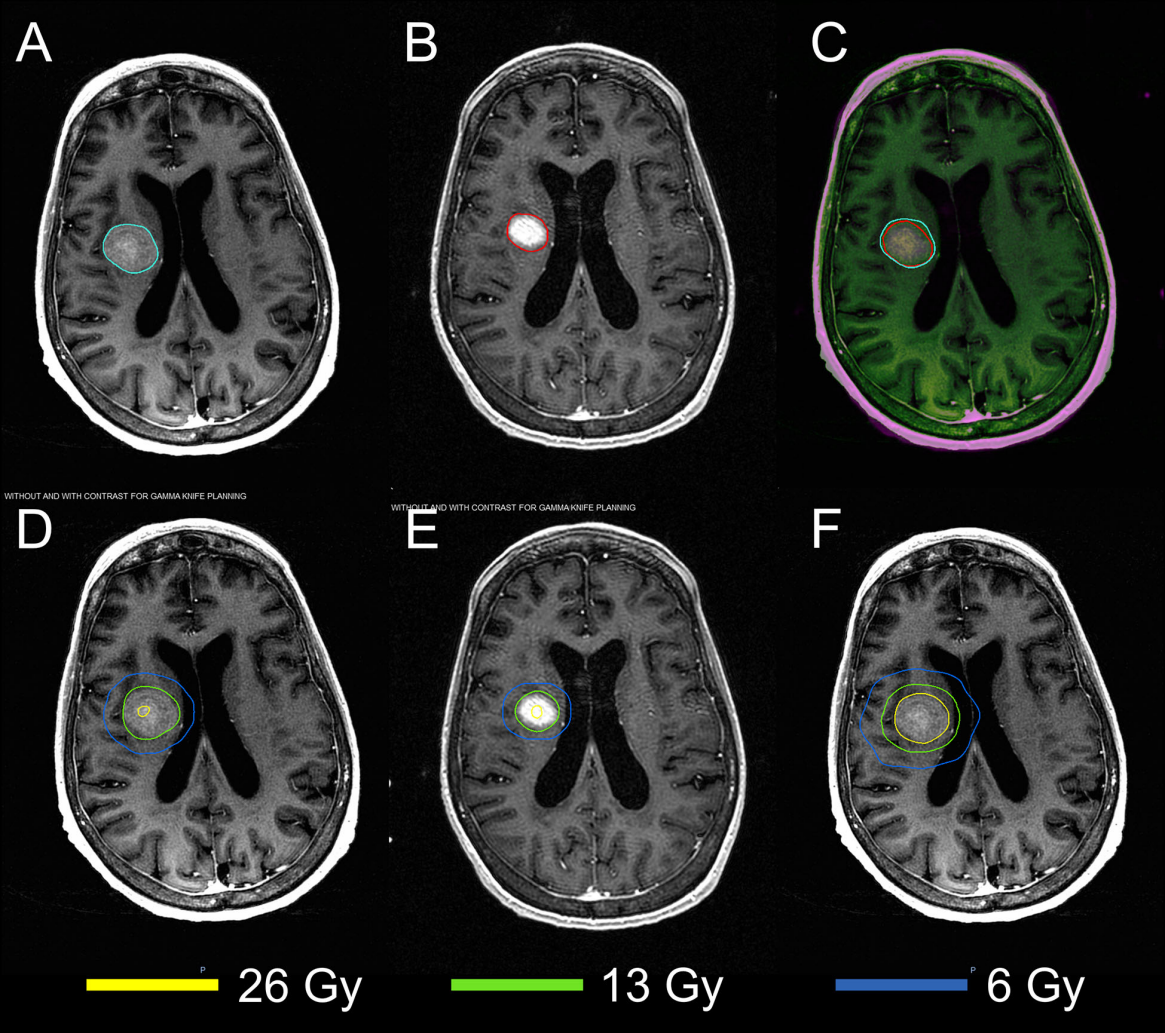
Demonstration of the dose summation for a representative case undergoing 2-stage GK SRS. Deformable image registration was performed to register the MRI acquired in the second session **(B)** to that acquired in the first session **(A)**. The registration accuracy was evaluated by visually examining the MRI fusion **(C)** and by computing the Dice similarity coefficient between the original tumor contour (cyan) on the first MRI and the deformed tumor contour (red) on the second MRI. With the same deformation, the original dose distribution in the second session **(E)** was deformed and added to that in the first session **(D)**. After the dose summation, thetotal dose distribution of the 2-stage GK SRS could be assessed on the first MRI **(F)**.

**Figure 3:**
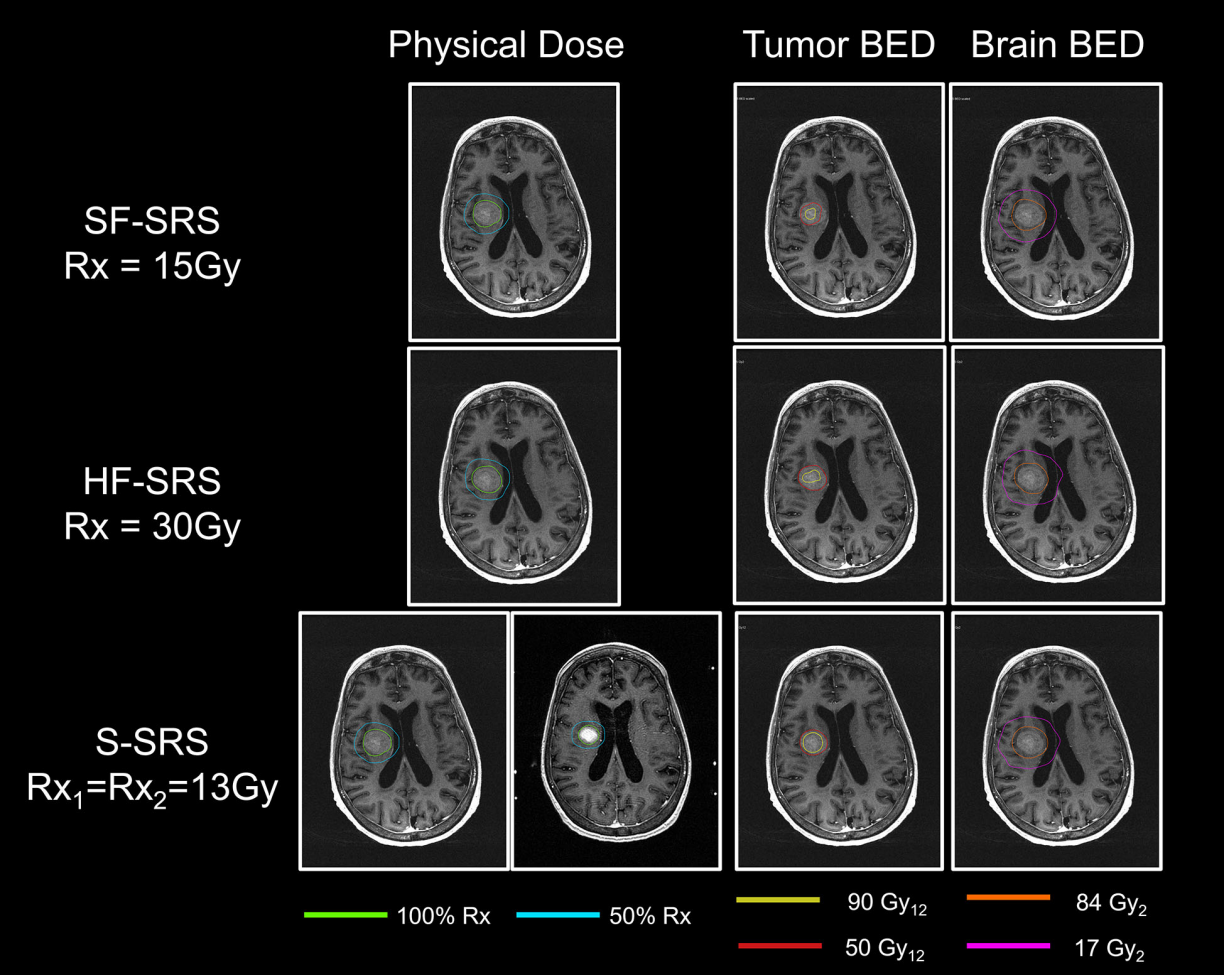
Comparisons of physical dose, total BED to tumor, and total BED to normal brain tissue between SF-SRS, HF-SRS, and Staged-SRS plans for a representative case. The isodose line of 100% and 50% of the prescription dose was shown. The total BEDs were displayed on the MRI acquired in the first session.

The MRIs acquired in two sessions were successfully registered using the DIR. **Figure 2** demonstrates the results of DIR and dose summation on a representative case. The DIR was performed to register the second MRI (**Figure 2B**) to the first MRI (**Figure 2A**). Although there were considerable anatomy changes between two sessions, e.g., the tumor shrunk by more than 50% from 7.92 cc to 3.60 cc, we were able to achieve reasonable registration results with the Dice similarity coefficient of 0.830, as shown in the fusion in **Figure 2C**. Across the 24 tumors, the median Dice similarity coefficient between the original and deformed tumor contours was 0.884 (range: 0.657–0.948), indicating the high accuracy of DIR between the MRIs. Ensured by the accurate registration, the dose delivered in the second session (**Figure 2E**) can be deformed and overlapped with that in the first session (**Figure 2D**), and the dose summation (**Figure 2F**) could be performed to determine the total physical dose delivered in both sessions. With the total physical dose in Staged-SRS, the BEDs to tumor and normal brain tissue were calculated and compared to those in SF-SRS and HF-SRS, as shown in **Figure 3**.

The results of BED in the Staged-SRS, SF-SRS, and HF-SRS plans were summarized in **Table 2**. We observed significant differences in the BED_98%_ of tumor between the Staged-SRS and SF-SRS plans (paired Wilcoxon; *p* < 0.001). Of the total 23 lesions, the mean BED_98%_ increased from 35.6 Gy_12_ in the SF-SRS plans (range: 27.1–47.2 Gy_12_) to 45.6 Gy_12_ in the Staged-SRS plans (range: 32.8–64.2 Gy_12_). However, there was no significant difference in V_84Gy2_ (paired Wilcoxon; *p* = 0.677) between the Staged-SRS (mean: 28.6 cc; range: 4.0–84.9 cc) and SF-SRS (mean: 28.3 cc; range: 4.0–80.8 cc) plans.

**Table 2:** Comparison of normal brain tissue V_84Gy2_ and tumor BED_98%_ between HF-SRS, SF-SRS, and Staged-SRS.

Patient	Normal Brain V_84Gy2_ (cc)			GTV BED_98%_ (Gy_12_)		
	HF-SRS	SF-SRS	Staged-SRS	HF-SRS	SF-SRS	Staged-SRS
1	6.7	9.5	7.4	51.8	32.2	43.7
2	8.6	10.6	10.8	50.4	27.1	32.8
3	7.1	9.4	8.2	53.0	35.9	45.0
4	36.7	45.3	42.9	50.5	35.9	42.5
				52.1	38.7	47.9
				50.9	36.2	41.8
5	71.0	75.2	78.5	49.6	34.9	34.4
				50.2	34.4	32.8
				51.5	33.7	35.6
				51.1	34.9	38.2
6	4.0	4.0	4.0	57.3	32.6	47.0
7	38.2	42.5	40.7	51.5	35.1	35.9
				51.4	35.7	33.2
				51.0	36.4	53.1
				51.6	36.2	49.2
8	65.0	80.8	84.9	51.5	36.0	56.7
				49.3	36.1	54.0
				48.8	33.8	52.3
				51.7	38.0	63.2
9	6.6	12.0	10.0	53.8	47.2	64.2
10	14.8	16.7	20.4	50.2	36.4	42.6
11	15.4	21.8	20.6	49.5	36.8	55.5
12	8.7	11.7	14.8	53.0	35.1	46.9

We did not observe any dosimetric advantages of the Staged-SRS over the HF-SRS plans. Higher BED could be delivered to tumor with significantly larger (paired Wilcoxon; *p* = 0.01) tumor BED_98%_ in the HF-SRS plans (mean: 51.4 Gy_2;_ range: 48.8–57.3 Gy_2_) compared to those in the Staged-SRS plans. Despite the additional 1-mm setup margin added to tumor and therefore the larger PTV in the HF-SRS plans, the V_84Gy2_ of the normal brain tissue in the HF-SRS plans (mean: 23.6 cc; range: 4.03–71.0 cc) remained significantly lower than those in the Staged-SRS plans (paired Wilcoxon; *p* < 0.001).

## Discussion

In this study, we presented an effective approach to quantitively evaluate BEDs to large BMs and OARs for GK Staged-SRS. The proposed approach applies DIR between the treatment MRIs to address the potential target volume shrinkage and the LQ model to account for tumor proliferation during the treatment interval for Staged-SRS. Since the implementations of DIR and the LQ model are independent of treatment modality, our method is therefore not limited to GK SRS and readily applicable to LINAC-based SRS as well. Our results showed that compared to SF-SRS, Staged-SRS had the dosimetric advantage of allowing higher BED delivered to tumor while maintaining comparable dose delivered to the normal brain tissue. However, despite irradiating larger PTVs due to the additional setup margin, HF-SRS delivered a lower dose to the normal brain tissue while maintaining higher BED to tumors.

DIR was implemented in our study to address the differences in patient anatomies between two Staged-SRS sessions due to tumor shrinkage. This approach has also been used to evaluate the composite BEDs in external beam radiotherapy when a patient undergoes multi-modality or multi-stage treatments to the same lesion (28, 29). Although this DIR-based method is widely used and well understood, the accuracy of DIR is crucial to ensure a biologically plausible dose summation and therefore needs to be cautiously verified (30). To deform an image to the reference image, the algorithm of DIR used in our study correlates pixels between two images based on the pixel values, which reflect the physical properties of the pixels. Therefore, to independently validate the DIR accuracy, we calculated and assessed the Dice similarity coefficient of the contours of the same target in the deformed and reference images, and the delineation of contours was based on not only the physical properties included in the pixel values but also the anatomical and clinical evidence.

There have been ongoing debates on the application of LQ model for SRS (31, 32). It was argued that the LQ model only considers radiation damage to tumor DNA, which results in cell death, while more evidence has shown that microvascular dysfunction and stromal damage could also trigger tumor cell death at a dose per fraction > 10 Gy based on the obliteration efficiency observed in arteriovenous malformation (AVM) data (33, 34). Because the LQ model fails to account for this endothelial cell apoptosis, Kirkpatrick et al. argued that the LQ prediction might not be appropriate for SRS and might underestimate tumor control (31). However, Brown et al. (35) rebutted that the currently available preclinical and clinical data (36, 37) are not sufficient to revoke the LQ model, and large BED delivered in SRS could still be correlated with the high tumor control rate since the standard radiobiology concepts of the 5 Rs still hold. Therefore, until a concrete alternative model is available, we still utilized the LQ model to assess the radiobiological effect of SRS in this study. We should also note that, because the LQ model was originally derived as a mathematical model to explain cell killing and was gradually extended to account for more radiobiological factors, the LQ model inevitably oversimplifies the complex nature of cell killing of radiation and does not address all the necessary factors. Therefore, the results derived from the LQ model should be interpreted with caution in clinical practice.

The total biologically equivalent dose delivered to a tumor in Staged-SRS is directly affected by the interval between the sessions of the Staged-SRS. As shown in Eq. 3, when the interval *T* is less than the tumor repopulation kick-off time *T_k_*, the proliferation of tumor cells could be ignored and the total dose to tumor is the summation of doses from each session. Once tumor cells start proliferating, the BED decreases at an approximate rate of 0.77 Gy_12_ per day. Therefore, a shorter interval is preferred to increase the total BED to tumor and therefore to boost the local control. However, a longer interval might also be advantageous to allow more tumor shrinkage and therefore to reduce normal brain tissue toxicities. In contrast to SF-SRS or HF-SRS where single or multiple radiation treatments are delivered in a shorter period of time and tumor volumes are usually considered as constant throughout the treatment course, the interval between the sessions in Staged-SRS allows tumors to shrink after being irradiated in the first session, as observed in both previous studies and our studies. This tumor shrinkage is beneficial to reduce the dose to normal brain tissue as it allows small tumor volume at the second session. Although there is yet a widely accepted model to evaluate tumor shrinkage after radiotherapy, we hypothesized that the tumor volume is inversely correlated with the interval time. Hence, as a compromise between higher total BED to tumor and smaller tumor volume, we suggest that the ideal interval should be equal to the tumor repopulation kick-off time, which is 28 days, and similar to what was used in previous Staged-SRS studies (10, 11, 38).

Our study is subject to several limitations. First, we did not distinguish different tumor pathologies when modeling the tumor cell repopulation during the session interval for Staged-SRS. It has been hypothesized that the proliferation rates may vary for tumor cells of different pathologies based on clinical observation. For example, head and neck cancer is known for its aggressive proliferation with a rapid clonogen repopulation rate (39–41), whereas the tumor repopulation of prostate cancer is usually much slower and similar to that of late-responding normal tissue (42–44). Because all the primary diseases of the BMs included in our study were fast-proliferating tumors and there is yet to be sufficient investigations on the tumor repopulation on each type of tumor, we deliberately ignored the heterogeneity in the tumor proliferation parameters *a,T_pot_* and *T_k_*. Second, in order to calculate the total BED delivered to tumor, we hypothesized that tumor volume remained unchanged throughout a treatment course of HF-SRS. However, more evidence is warranted to support this hypothesis. Had daily image study become available for HF-SRS, we might have observed different tumor volumes throughout a treatment course, which could have resulted in different tumor BEDs compared to Staged-SRS. Furthermore, several patients in our study started the second sessions after the ideal interval due to logistical issues, which resulted in sub-optimal BED delivered to the tumors. For example, the longest interval observed in our study was 51 days. Had the patient been treated 28 days after the first session, the BED to each tumor in the Staged-SRS would have increased by 17.7 Gy_12_ and the BED_98%_ of each tumor would have been comparable to those in the HF-SRS plans. Finally, the purpose of this study is to provide a theoretic tool to evaluate the BED to both tumor and normal tissue to assist plan evaluation. Given the lack of consensus on the adaptability of the LQ model for SRS and the approximated model parameters used in the proposed approach, future clinical investigations are essential to further validate the model and to fine-tune the model parameters. Until then, caution needs to be exercised in using the presented data for clinical decision-making purposes.

## Conclusion

We presented an effective approach to calculate the composite BEDs delivered to both tumor and normal brain tissue for Staged-SRS. Compared to SF-SRS, Staged-SRS is a more effective treatment option in patients with large BMs with a higher dose to tumor and a comparable dose to normal brain tissue. However, compared to HF-SRS, Staged-SRS is not dosimetrically superior with a lower dose to tumor but with a higher dose to normal brain tissue.

## Data Availability Statement

The original contributions presented in the study are included in the article/supplementary material. Further inquiries can be directed to the corresponding author.

## Ethics Statement

The studies involving human participants were reviewed and approved by Rutgers University Institutional Review Board. Written informed consent for participation was not required for this study in accordance with the national legislation and the institutional requirements.

## Author Contributions

TC designed the study, analyzed the data, and wrote the manuscript. TC, JW, SD, and KN led the conception and design of the study. TC and KN generated the clinical treatment plans. JW, SD, AC, and NO provided patient data and reviewed the treatment plans, TC, SD, AC, NY, and KN revised the article. All authors contributed to the article and approved the submitted version.

## Conflict of Interest

The authors declare that the research was conducted in the absence of any commercial or financial relationships that could be construed as a potential conflict of interest.

## Publisher’s Note

All claims expressed in this article are solely those of the authors and do not necessarily represent those of their affiliated organizations, or those of the publisher, the editors and the reviewers. Any product that may be evaluated in this article, or claim that may be made by its manufacturer, is not guaranteed or endorsed by the publisher.
